# First-principles investigation of possible room-temperature topological insulators in monolayers

**DOI:** 10.1039/d3ra05619h

**Published:** 2023-10-27

**Authors:** Alina Chen, Xuan Luo

**Affiliations:** a National Graphene Research and Development Center Springfield Virginia 22151 USA

## Abstract

A Quantum Spin Hall (QSH) insulator with a large bulk band gap and tunable topological properties is crucial for both fundamental research and practical application. Chemical function-alization has been proposed as an effective route to realize the QSH effect. Using the ABINIT package, we have investigated the properties of (1) TlP, the functionalized monolayers TlPX_2_ (X = F, Cl, Br, I); (2) TlAs, the functionalized monolayers TlAsX_2_ (X = F, Cl, Br, I), and (3) GaGeTe, InGeTe, and InSnTe systems. The topological nature is verified by the calculation of the *Z*_2_ topo-logical invariant. We discovered TlPF_2_, TlPCl_2_, TlPBr_2_, TlPI_2_, TlAs, TlAsF_2_, TlAsCl_2_, TlAsBr_2_, and TlAsI_2_ were promising 2D TIs with bulk band gaps as large as 0.21 eV. Each monolayer was suitable for room-temperature application, and show great potential for their future applications in quantum computers, nanoelectronics, and spintronics.

## Abstract 2

Several promising 2D topological insulators are identified using ABINIT. Key monolayers show robust band gaps, indicating immense potential for applications in physics and chemistry.

## Graphical abstract

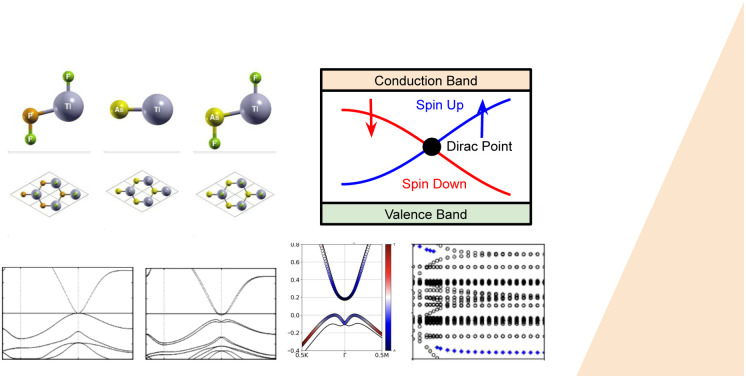

## 1. Introduction

Topological insulators (TIs) are a class of materials that have created a surge of research activities in the past decade.^1–4^ Due to their intriguing properties and promising applications in spintronics and quantum computations, they are attracting worldwide interest.^5–8^ The term “topological insulator” was coined by Moore and Balents in their paper to propose the existence of TIs in 3-Dimensional (3D) systems,^1,9^ but their story started long before the discovery of 3D TIs. The Quantum Hall System, discovered in 1980, is considered to be the first TI that became known to physicists.^1^ The Spin Hall Effect was experimentally confirmed by Kato *et al.* in 2004,^1,10^ which led Murakami, Nagaosa, and Zhang to propose the idea of a Spin Hall Insulator,^11^ a gapped insulator with zero charge conductivity but with a finite spin Hall conductivity due to a finite Berry phase of the occupied states.^12^ Although this proposal could not generate spin currents in the absence of any electrons at the Fermi level, it triggered Kane and Mele's proposal of its quantized version, the Quantum Spin Hall (QSH) insulator.^1,13^

Many materials have been predicted to be TIs, but only some have been addressed ex-perimentally. The first material that was experimentally identified as a time-reversal (TR) invariant TI was the CdTe/HgTe/CdTe quantum well,^1,14^ or a thin layer of HgTe sand-wiched by CdTe. Afterwards, the AlSb/InAs/GaSb/AlSb quantum well was theoretically predicted^15^ and experimentally confirmed^16,17^ to be a 2D TI system. The first 3D TI material that was experimentally identified was Bi_1−*x*_Sb_*x*_,^18^ an alloy of Bi and Sb. Discoveries of both 2D and 3D TI materials are ongoing and strongly called for. However, 2D TIs have both an insulating bulk and conducting edge states that display unique advantages over 3D TIs with regard to flexibility, a higher charge carrier mobility, and controllability owing to their atomic thickness.^5^

The last decade has seen an immense development of interest in monolayer materials.^19^ A monolayer forms when the thickness of material is reduced down to a single atom.^1^ A common feature of materials that show band structures with room temperature 2D TI properties are they most likely have 2D hexagonal honeycomb-like crystal structures, indicating that a 2D hexagonal lattice could be an excellent cradle to breed QSH insulators with the influence of spin–orbit coupling (SOC).^5^ TlX (X = N, P, As, Sb) monolayers were recently reported to possess structural stability.^5,19^ First-principles calculations confirmed that a two-dimensional TlP monolayer could convert into a topological insulator with the effect of bromination accompanied by a large bulk band gap of 76.5 meV, which meets the requirement for room-temperature application.^20^

GaGeTe, InSnTe, and InGeTe were promising monolayers due to GaGeTe's high car-rier mobility and tunable band structure.^21^ Each monolayer has a layered crystal structure stacked from six-atom thick building blocks. First-principles calculations have identified the GaGeTe-type periodic structures as a potential host for topological phases.^22^ The layered In–SnTe bulk material is predicted to be a 3D strong topological insulator with *Z*_2_ = 1; (111).^22^

Additionally, the tetrahedral atomic coordination in the GaGeTe-type structures closely resembles the topological materials with the diamond-like cubic lattices.^14,23,24^ Possible ways to induce topological order could be doping GaGeTe with larger isovalent p-elements, such as In and Sn.

The crucial bottleneck of reported 2D TIs is their small bulk band gaps, which are too weak to be reflected in modern experimental conditions. Graphene, for example, was the first material predicted to realize a TI, but the gap was unobservably small due to carbon's weak spin-orbital coupling.^25^ A large bulk band gap is crucial for protecting the edge current against the interference of thermally activated carriers.^5^ Fortunately, chemical functionalization of 2D materials is a powerful tool for creating new materials with desirable features.^26^ Chemical functionalization of topological insulator monolayers is an effective method of tuning the band gap, while preserving the nontrivial topological order.^27–29^ For example, pristine stanene has a band gap of 0.1 eV, but with functional groups, the band gap reaches 0.3 eV.^27^ This research studies possible 2D TIs by performing first-principles calculations on both monolayers and chemically functionalized monolayers. We performed calculations on the monolayers TlP and TlAs, which were chemically functionalized with halogens, and on the monolayers GaGeTe, InSnTe, and InGeTe. These findings may endow the monolayers with the potential to fabricate new quantum devices operating at room temperature in nanoelectronics and spintronics.

## 2. Method

We performed first-principle calculations based on Density Functional Theory (DFT) using the Generalized Gradient Approximation (GGA) exchange–correlation in the Perdew–Burke–Ernzerhof (PBE)^30^ format implemented in the ABINIT^31,32^ code. We use the Projected Augmented Wave (PAW) method^33^ with projectors generated using the AtomPAW code.^34,35^ The electron configurations and radial cutoffs used to generate the PAW pseudopotentials are shown in Table 1.

**Table 1:** Electron configurations and radial cutoffs used to generate the PAW pseudopotentials for the elements used in this study

Element	Atomic number	Electron configuration	Radius cutoff (Bohr)
Tl	81	[Xe 4f^14^] 6s^2^ 6p^1^ 5d^10^	2.42
P	15	[Ne] 3s^2^ 3p^3^	1.91
As	33	[Ar] 4s^2^ 4p^3^ 3d^10^	2.10
F	9	[He] 2s^2^ 2p^5^	1.40
Cl	17	[Ne] 3s^2^ 3p^5^	1.80
Br	35	[Ar 3d^10^] 4s^2^ 4p^5^	2.20
I	53	[Kr 4d^10^] 5s^2^ 5p^5^	2.30
Ga	31	[Ar] 4s^2^ 4p^1^ 3d^10^	2.10
Ge	32	[Ar] 4s^2^ 4p^2^ 3d^10^	2.30
Te	52	[Kr 4d^10^] 5s^2^ 5p^4^	2.31
In	49	[Kr] 5s^2^ 5p^1^ 4d^10^	2.51
Sn	50	[Kr] 5s^2^ 5p^2^ 4d^10^	2.51

In total energy calculations, self-consistent cycles were recognized when the total energy difference was less than 1.0 × 10^−5^ Hartree twice consecutively. The kinetic energy cutoff, the Monkhorst–Pack grid, and the vacuum height of the unit cell were converged for each monolayer. The converged values corresponding to each monolayer were used for calculations with and without SOC.

The Broyden–Fletcher–Goldfarb–Shanno (BFGS) minimization algorithm was used to perform structural optimization. The atomic structure was relaxed until the maximum atomic forces were less than 5.0 × 10^−5^ Hartree Bohr^−1^. The relaxed structure corresponding to each monolayer was used for band structure calculations with and without SOC.

The *Z*_2_ topological invariant was computed by tracking the evolution of the hybrid Wannier charge centers by implementing the Wannier 90 package^36,37^ and the Z2Pack software package.^38–40^ The Wannier charge centers are based on the notion of Wannier orbitals.

These are given by Fourier transforming the Bloch States1
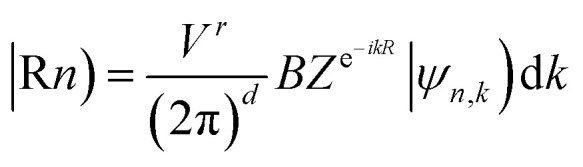
where *d* is the dimensionality of the system and *V* is the unit cell volume. These orbitals can be changed by a Gauge transformation which affects their localization and position in real space. To compute topological invariants, hybrid Wannier orbitals are introduced: they are Fourier transforms performed only in one spatial direction, for example2
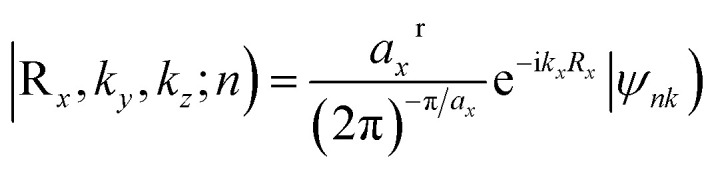


## 3. Results and discussion

The atomic structure, band structures with and without SOC, and *Z*_2_ topologies of each monolayer will be described for TlP, TlAs, GaGeTe, InGeTe, InSnTe, and the chemically functionalized monolayers TlPX_2_ and TlAsX_2_, where X = F, Cl, Br, and I. The band structures were calculated using the high-symmetry *k*-points *M* (1/2, 1/2, 0.0), *K* (2/3, 1/3, 0.0), 2 2 3 3 and *Γ* (0.0, 0.0, 0.0).

### 3.1. TlPX_2_ (X = F, Cl, Br, I)

**Fig. 1:**
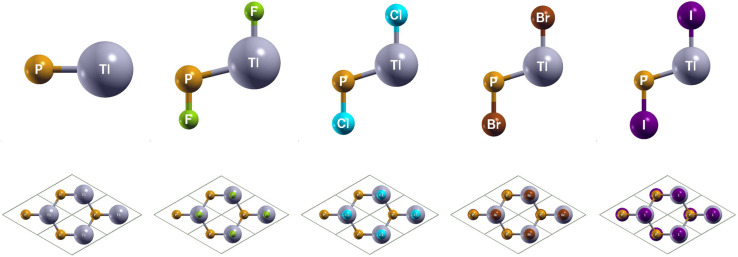
Optimized atomic structures of TlP and TlPX_2_ (X = F, Cl, Br, I) are shown from left to right. The atomic structures can be identified by the labels on each atom. Here, gray, orange, green, cyan, brown, and purple represent Tl, P, F, Cl, Br, and I atoms, respectively.

The side and top views of the atomic structures are presented in Fig. 1. The monolay-ers have a honeycomb lattice. Similar to silicene, the Tl and P atoms occupy two sublattices with a buckled height of 1.14 Bohr and a lattice constant of 8.14 Bohr. It is characterized by the sp^2^ hybridization. The calculated lattice constants and bond lengths of each monolayer can be found in Table 2. When TlP is chemically functionalized, each Tl (P) atom is bonded to a X atom and three P (Tl) atoms, which is analogous to a typical III–V bulk counterpart. Thus, the sp^3^ hybridization is naturally formed, leading to an increase in the buckled height and bond lengths. Our results confirm that chemically functionalizing TlP leads to a consistent increase in each monolayer's buckled height, in agreement with previous results.^20^ The buckled height increased by 0.1 Bohr, 0.36 Bohr, 0.39 Bohr, and 0.39 Bohr respectively for the monolayers TlPF_2_, TlPCl_2_, TlPBr_2_, and TlPI_2_. A similar phenomenon has been observed in previous research.^20^ The bond length Tl–P increased from 4.84 Bohr for the monolayer TlP to 5.12 Bohr, 5.11 Bohr, 5.12 Bohr, and 5.16 Bohr for the chemically functionalized monolayers TlPF_2_, TlPCl_2_, TlPBr_2_, and TlPI_2_, respectively.

**Table 2:** Calculated optimized structural parameters of TlP and TlPX_2_ (X = F, Cl, Br, I): the lattice constant (*a*) in Bohr, bond length P − X (d_P−X_) in Bohr, bond length Tl − P (d_Tl−P_) in Bohr, bond length Tl − X (d_Tl−X_) in Bohr, and buckled height (d_b_) in Bohr. A dash denotes the information is not applicable. Band gaps and topologies of TlP and TlPX_2_, including the band gap without SOC *E*_g_ in electron-volts and the band gap with SOC *E*_g-SOC_ in electron-volts. d and id stand for direct and indirect band gap, respectively. *Z*_2_ is the topological index

System	*a* (Bohr)	d_P-X_ (Bohr)	d_Tl−P_ (Bohr)	d_Tl−X_ (Bohr)	d_b_ (Bohr)	*E*_g_ (eV)	*E*_g-SOC_ (eV)	*Z*_2_
TlP	8.14	—	4.84	—	1.14	0.23 (d)	0.18 (d)	0
TlPF_2_	8.60	3.07	5.12	3.90	1.24	0.00 (d)	0.04 (id)	1
TlPCl_2_	8.46	3.89	5.11	4.60	1.50	0.00 (d)	0.03 (id)	1
TlPBr_2_	8.46	4.22	5.12	4.87	1.53	0.00 (d)	0.07 (id)	1
TlPI_2_	8.54	4.65	5.16	5.27	1.53	0.00 (d)	0.15 (id)	1

**Fig. 2:**
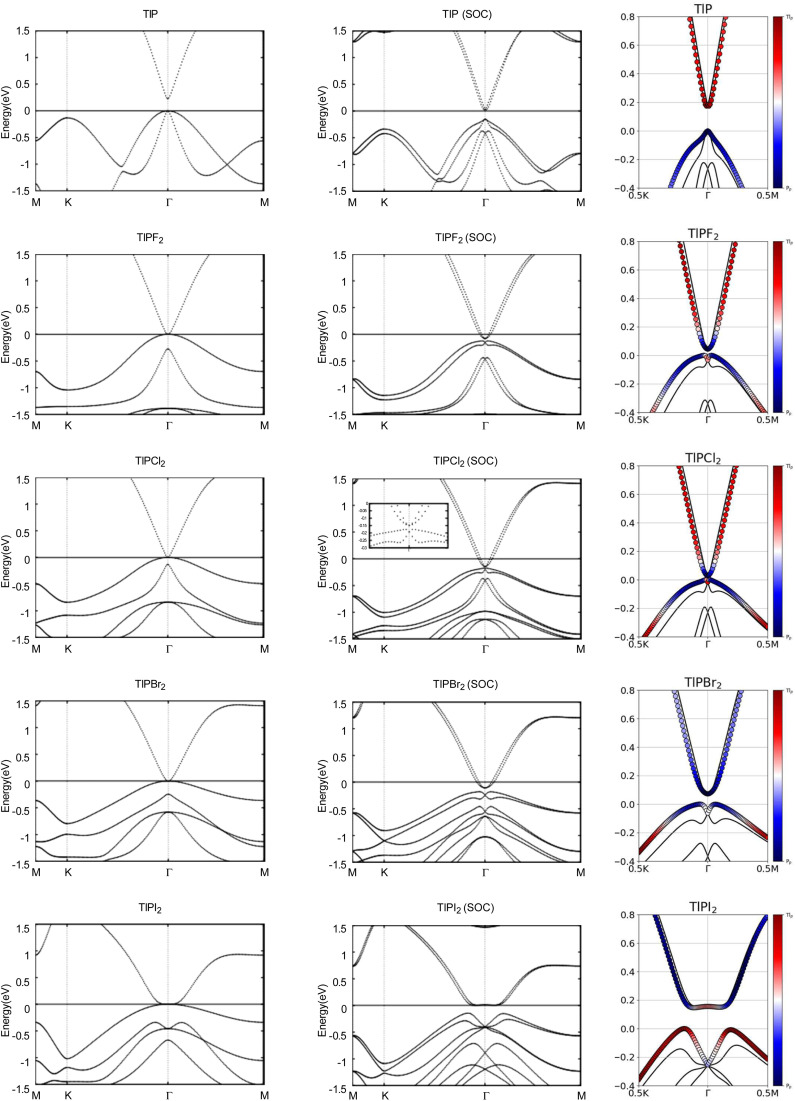
Calculated band structures with and without SOC of TlP and TlPX_2_ (X = F, Cl, Br, I), with the orbital-resolved band structures with SOC. The Fermi energy is set to 0 eV. The red and blue denote contributions from the 6p and 3p orbitals, respectively. The band structures can be identified by their titles.

Next, we turn towards the electronic properties of TlP and the chemically functionalized TlP monolayers. The band structures with and without an SOC effect are displayed in Fig. 2. Without applying SOC, TlP is a semiconductor with a small direct band gap of 0.23 eV at the *Γ* point. TlPF_2_, TlPCl_2_, TlPBr_2_, and TlPI_2_ have zero energy band gaps with the valence band maximum (VBM) and the conduction band minimum (CBM) degenerate at the *Γ* point, forming a single Dirac point. The band gaps are displayed in Table 2.

Typically, the effect of SOC on the electronic structures of semiconductors is negligible.^41^ However, it is crucial for the case of semimetals or metallic systems. When SOC is taken into account, TlPF_2_, TlPCl_2_, TlPBr_2_, and TlPI_2_ have indirect energy gaps of 0.04 eV, 0.03 eV, 0.07 eV, and 0.15 eV, respectively. Unlike the chemically functionalized monolayers, TlP's valence band and conduction band appear relatively closer together with SOC and the band gap decreases from 0.23 eV to 0.18 eV. The band gaps of TlP and the chemically functionalized monolayers, TlPF_2_, TlPCl_2_, TlPBr_2_, and TlPI_2_, most likely change dramatically due to the strong SOC of the Tl and P atoms.^5^ The SOC-induced energy gaps are larger than the thermal energy at room temperature (0.026 eV), implying that the band gaps for each monolayer would be feasibly measured at room temperature.

The band structures of TlP and the TlPX_2_ monolayers are projected on the p orbitals of the elements. These band structures can be found in the third column of Fig. 2. Band inversion is absent in TlP, and present in some of the other chemically functionalized TlP monolayers. Band inversion has long been cited as a strong indicator of a topological insulator.^20,41^. However, our results confirm the prediction that band inversion is conceptually not a necessary outcome of topological phase transitions.^42^

**Fig. 3:**
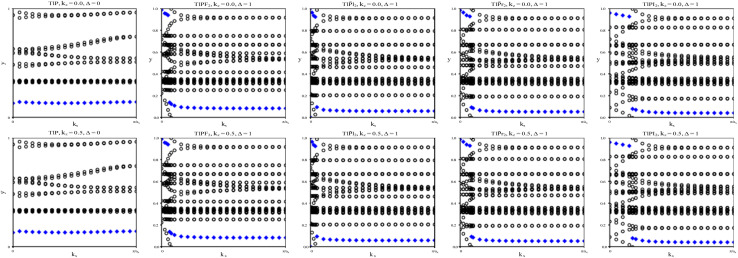
The trajectories of the Wannier charge centers for TlP and TlPX_2_ (X = F, Cl, Br, I). The open circles represent hybrid Wannier charge centers. The first row is at the surface *k*_*z*_ = 0. The second row is at the surface *k*_*z*_ = 0.5. The plots can be identified by their titles.

To verify our curiosities about the QSH state in the chemically functionalized TlP monolayers, the *Z*_2_ topological invariant is an important decision criteria for a material's classification as a TI.^20^ We directly calculate the *Z*_2_ topological invariants and list them in Table 2. Fig. 3 shows the trajectories of the Wannier charge centers at the surfaces *k*_*z*_ = 0 and *k*_*z*_ = 0.5. The hybrid Wannier charge centers (HWCC) form bands, similar to the band structure of a dispersion relation. The *Z*_2_ index can be calculated using the Wilson loop using the evolution of the Wannier charge centers. We calculate the *Z*_2_ invariants on a coarse k-mesh, considering the points marking the middle of the largest gap at each *k*_*x*_. These points are marked by blue diamonds on Fig. 3. Whenever the location of the middle of the gap changes between two adjacent *k*_*x*_ values, we count the number of HWCC that exist between the two gap centers and sum this number for all the crossings as *k*_*x*_ goes from 0 to π. If this value is even, *Z*_2_ = 0, and if its odd, *Z*_2_ = 1. The *Z*_2_ calculations confirm that TlPF_2_, TlPCl_2_, TlPBr_2_, and TlPI_2_ are promising 2D TIs, with *Z*_2_ = 1, while TlP is a normal insulator, with *Z*_2_ = 0. Our results for TlP corroborate Li *et al.*'s calculations with the plane-wave basis Vienna *ab initio* simulation package (VASP).^5^

### 3.2. TlAsX_2_ (X = F, Cl, Br, I)

**Fig. 4:**
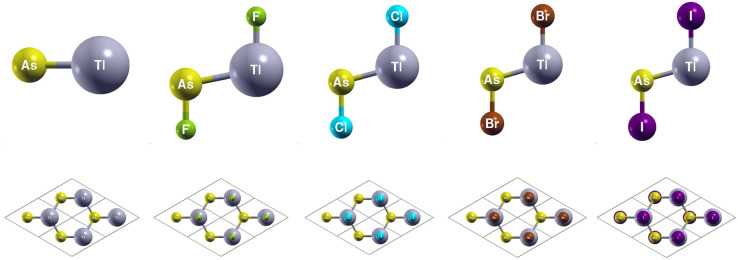
Optimized atomic structures of TlAs and TlAsX_2_ (X = F, Cl, Br, I) monolayers are shown from left to right. The atomic structures can be identified by the labels on each atom. Here, gray, yellow, green, cyan, brown, and purple represent Tl, P, F, Cl, Br, and I atoms, respectively.

You can find the side and top views of the atomic structures in Fig. 4. The monolayers have a honeycomb lattice. Similar to TlP, the Tl and As atoms occupy two sublattices with a buckled height of 1.32 Bohr and a lattice constant of 8.14 Bohr. The calculated lattice constants and bond lengths of each monolayer can be found in Table 3. When TlAs is chemically functionalized, each Tl (As) atom is bonded to a X atom and three As (Tl) atoms. Our results confirm that chemically functionalizing TlAs leads to a consistent increase in each monolayer's buckled height for the monolayers TlAsCl_2_, TlAsBr_2_, and TlAsI_2_. The buckled height increased by 0.08 Bohr, 0.17 Bohr, and 0.12 Bohr, respectively. However, the buckled height decreased by 0.25 Bohr for the monolayer TlAsF_2_. This may be due to the difference in electronegativities, as the electronegativity of F is much higher than Cl, Br, or I. When As is functionalized with a more electronegative atom, there can be a stronger pull on the electrons. Because As is one period below P, it is heavier and has more electron shielding, leading to the changes in geometric structure. The bond length Tl–As increased from 5.06 Bohr for the monolayer TlAs to 5.32 Bohr, 5.31 Bohr, 5.31 Bohr, and 5.34 Bohr for the chemically functionalized monolayers TlAsF_2_, TlAsCl_2_, TlAsBr_2_, and TlAsI_2_, respectively. Previous literature has concluded that TlAs has a buckled height of 1.40 Bohr and a lattice constant of 8.54 Bohr.^5,20^

**Table 3:** Calculated optimized structural parameters of TlAs and TlAsX_2_ (X = F, Cl, Br, I): the lattice constant (*a*) in Bohr, bond length As − X (d_As−X_) in Bohr, bond length Tl − As (*d*_Tl−As_) in Bohr, bond length Tl − X (*d*_Tl−X_) in Bohr, and buckled height (d_b_) in Bohr. A dash denotes the information is not applicable. Band gaps and topologies of TlAs and TlAsX_2_, including the band gap without SOC *E*_g_ in electron-volts and the band gap with SOC *E*_g-SOC_ in electron-volts. d and id stand for direct and indirect band gap, respectively. *Z*_2_ is the topological index

System	*a* (Bohr)	d_As−X_ (Bohr)	d_Tl−As_ (Bohr)	d_Tl−X_ (Bohr)	d_b_ (Bohr)	*E*_g_ (eV)	*E*_g-SOC_ (eV)	*Z*_2_
TlAs	8.14	—	5.06	—	1.32	0.01 (d)	0.14 (id)	1
TlAsF_2_	8.60	3.35	5.32	3.93	1.07	0.00 (d)	0.18 (id)	1
TlAsCl_2_	8.46	4.13	5.31	4.63	1.40	0.00 (d)	0.15 (id)	1
TlAsBr_2_	8.46	4.43	5.31	4.90	1.49	0.00 (d)	0.18 (id)	1
TlAsI_2_	8.54	4.86	5.34	5.35	1.44	0.00 (id)	0.21 (id)	1

**Fig. 5:**
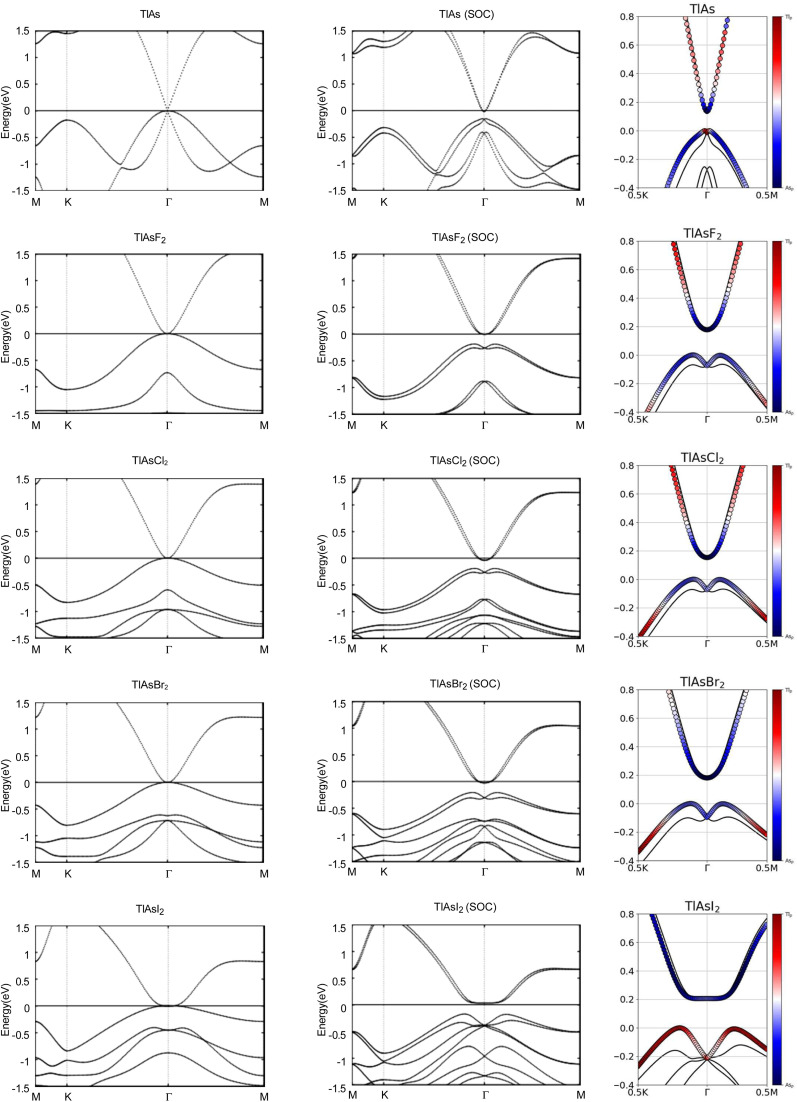
Calculated band structures with and without SOC of TlAs and TlAsX_2_ (X = F, Cl, Br, I), with the orbital-resolved band structures with SOC. The Fermi energy is set to 0 eV. The red and blue denote contributions from the 6p and 4p orbitals, respectively. The band structures can be identified by their titles.

Next, we investigated the electronic properties of TlAs and the chemically functionalized TlAs monolayers. The band structures with and without an SOC effect are displayed in Fig. 5. Without applying SOC, TlAs has a small direct band gap of 0.01 eV at the *Γ* point. TlAsF_2_, TlAsCl_2_, and TlAsBr_2_ have zero energy band gaps TlAsI_2_ has a zero energy indirect band gap, with the VBM and CBM degenerate at the *k*-points (0.0402, 0.0402, 0.0000) and (0.0345, 0.0345, 0.0000), respectively. The band gaps are displayed in Table 3.

When SOC is taken into account, TlAs, TlAsF_2_, TlAsCl_2_, TlAsBr_2_, and TlAsI_2_ have indirect energy gaps of 0.14 eV, 0.18 eV, 0.15 eV, 0.18 eV, and 0.21 eV, respectively. The band gaps of TlAs, TlAsF_2_, TlAsCl_2_, TlAsBr_2_, and TlAsI_2_ most likely change dramatically due to the strong SOC of the Tl and As atoms. The SOC-induced energy gaps are much larger than the thermal energy at room temperature (0.026 eV), implying that the band gaps for each monolayer would be feasibly measured at each room temperature.

The band structures of TlAs and the TlAsX_2_ monolayers are projected on the p orbitals of the elements, similar to the TlP and the TlPX_2_ monolayers. These band structures can be found in the third column of Fig. 5. Band inversion is present in some of the chemically functionalized TlAs monolayers.

**Fig. 6:**
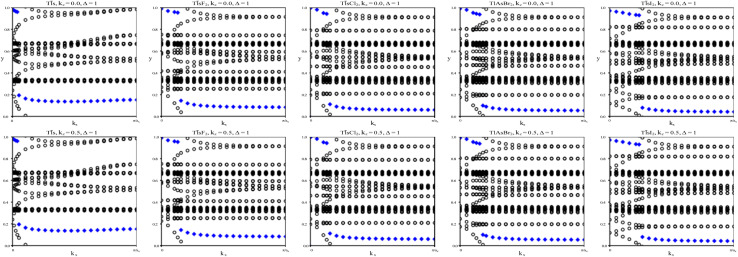
The trajectories of the Wannier charge centers for TlAs and TlAsX_2_ (X = F, Cl, Br, I). The open circles represent hybrid Wannier charge centers. The first row is at the surface *k*_*z*_ = 0. The second row is at the surface *k*_*z*_ = 0.5. The plots can be identified by their titles.

We calculate the *Z*_2_ topological invariants and list them in Table 3. Fig. 6 shows the trajectories of the Wannier charge centers at the surfaces *k*_*z*_ = 0 and *k*_*z*_ = 0.5. The *Z*_2_ invariants are calculated identical to how they were calculated for TlP and the chemically functionalized TlP monolayers. The *Z*_2_ calculations confirm that TlAsF_2_, TlAsCl_2_, TlAsBr_2_, and TlAsI_2_ are promising 2D TIs with *Z*_2_ = 1. Although TlAs has a nonzero band gap of 0.01 eV without SOC, it is less than the thermal energy at room temperature (0.026 eV), and is therefore insignificant and does not affect our results. Because its *Z*_2_ topological invariant is equal to 1, we can strongly conclude that TlAs is a promising 2D TI. Our results for TlAs corroborate Li *et al.*'s calculations with the plane-wave basis Vienna *ab initio* simulation package (VASP).

### 3.3. GaGeTe, InGeTe, InSnTe

**Fig. 7:**
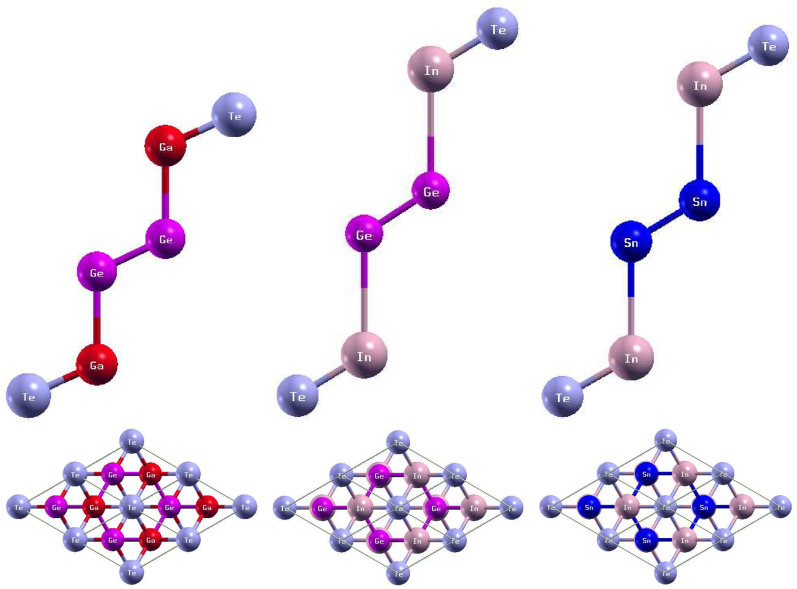
Optimized atomic structures of the GaGeTe, InGeTe, and InSnTe monolayers are shown from left to right. The atomic structures can be identified by the labels on each atom. Here, magenta, red, violet, and pink atoms represent Ge, Ga, Te, and In atoms, respectively.

The side and top views of the atomic structures are presented in Fig. 7. The monolayers have buckled honeycomb atomic arrangements.^43^ When thinned from bulk to monolayer, GaGeTe undergoes a transition from semimetal to semiconductor.^21^ The calculated lattice constants and bond lengths of each monolayer can be found in Table 4. GaGeTe, InGeTe, and InSnTe have calculated lattice constants of 8.14 Bohr, 8.60 Bohr, and 8.47 Bohr, respectively. After substituting In atoms with the Ga atoms of GaGeTe, the bond length Te − In was 0.59 Bohr larger than the bond length Te − Ga. The bond length In − Ge was 0.31 Bohr larger than the bond length Ga − Ge. The bond length Ge − Ge increased by 0.05 Bohr. After substituting Sn atoms with the Ge atoms of InGeTe, the bond length Te − In increased by 0.13 Bohr. The bond length In − Sn was 0.34 Bohr larger than the bond length In − Ge. The bond length Sn − Sn was 0.43 Bohr larger than the bond length Ge − Ge. Previous literature has concluded that GaGeTe has a lattice constant of 7.41 Bohr, in disagreement with the calculated lattice constant of 8.14 Bohr.^21,44,45^

**Table 4:** Calculated optimized structural parameters of GaGeTe, InGeTe, and InSnTe: the lattice constant (*a*) in Bohr, bond length Te − M (d_Te−M_) in Bohr where M = Ga, In, bond length

System	*a* (Bohr)	d_Te−M_ (Bohr)	d_M−N_ (Bohr)	d_N−N_ (Bohr)	*E*_g_ (eV)	*E*_g−SOC_ (eV)	*Z*_2_
GaGeTe	8.14	4.80	4.72	4.83	0.87 (d)	0.74 (d)	0
InGeTe	8.60	5.39	5.03	4.88	0.08 (id)	0.02 (id)	0
InSnTe	8.47	5.52	5.37	5.31	0.24 (d)	0.07 (d)	1

**Fig. 8:**
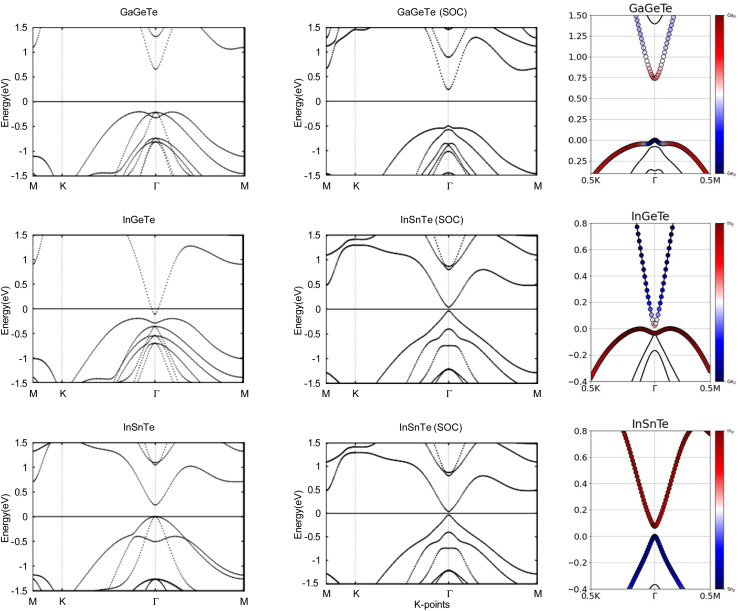
Calculated band structures with and without SOC of GaGeTe, InGeTe, and InSnTe, with the orbital-resolved band structures with SOC. The Fermi energy is set to 0 eV. The red and blue denote contributions from the 4p and 4p orbitals, 5p and 4p orbitals, and 5p and 5p orbitals, respectively. The band structures can be identified by their titles.

Next, we turn towards the electronic properties of GaGeTe, InGeTe, and InSnTe. The band structures with and without an SOC effect are displayed in Fig. 8. Without applying SOC, GaGeTe and InSnTe are semiconductors with direct band gaps of 0.87 eV and 0.24 eV at the *Γ* point, respectively. InGeTe is a semiconductor with a small indirect band gap of 0.08 eV, with the VBM at (0.1200, 0.0600, 0.0000) and the CBM at (0.0000, 0.0000, 0.0000).

The band gaps are displayed in Table 4.

When SOC is taken into account, GaGeTe and InSnTe have direct energy gaps of 0.74 eV and 0.07 eV. With SOC, InGeTe has an indirect energy gap of 0.02 eV, with the VBM at (0.0690, 0.0690, 0.0000) and the CBM at (0.0000, 0.0000, 0.0000). All three of these energy gaps decreased by 0.13 eV, 0.06 eV and 0.17 eV, respectively, from the energy gaps without SOC. This behavior opposes TlPX_2_ and TlAsX_2_.

The band structures of the GaGeTe, InGeTe, and InSnTe monolayers are projected on the p orbitals of the elements. These band structures can be found in the third column of Fig. 8. Band inversion is present in some of the monolayers.

Although we are inclined to believe that a QSH state does not exist in each GaGeTe, M − N (d_M−N_) in Bohr where N=Ge, Sn, and bond length N − N (d_N−N_) Bohr. Band gaps and topologies of GaGeTe, InGeTe, and InSnTe, including the band gap without SOC *E*_g_ in electron-volts and the band gap with SOC *E*_g-SOC_ in electron-volts. d and id stand for direct and indirect band gap, respectively. *Z*_2_ is the topological index.

**Fig. 9:**
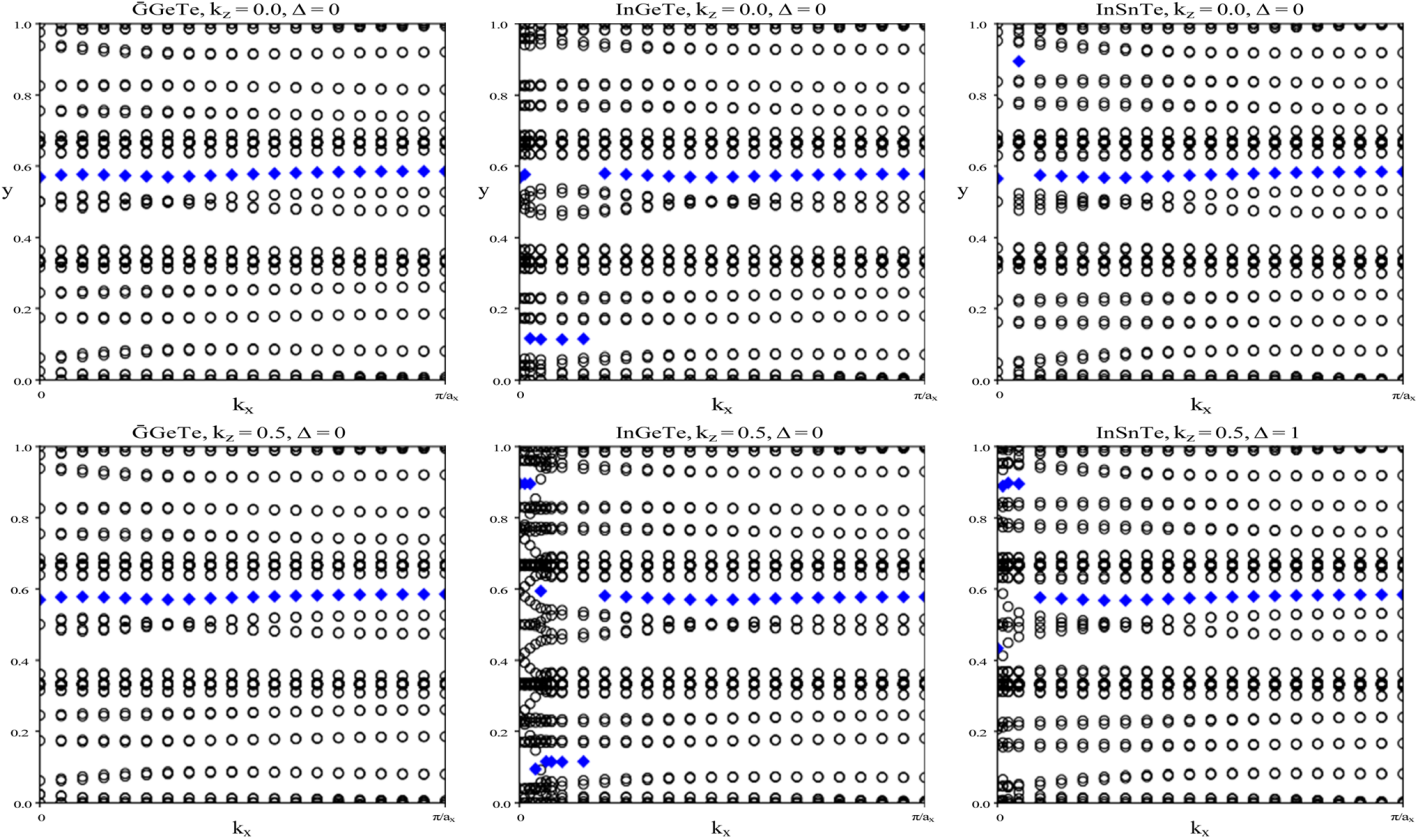
The trajectories of the Wannier charge centers for GaGeTe, InGeTe, and InSnTe. The open circles represent hybrid Wannier charge centers. The first row is at the surface *k*_*z*_ = 0. The second row is at the surface *k*_*z*_ = 0.5. The plots can be identified by their titles.

InGeTe, and InSnTe monolayer, we directly calculate the *Z*_2_ topological invariants and list them in Table 4. The trajectories of the Wannier charge centers can be found in Fig. 9 at the surfaces *k*_*z*_ = 0 and *k*_*z*_ = 0.5. The *Z*_2_ calculations confirm that GaGeTe and InGeTe are normal insulators, with *Z*_2_ = 0. Even though bulk GaGeTe has been investigated as a topological semimetal,^43^ the GaGeTe monolayer does not have topological properties. Although it is true that band inversion can be seen in the GaGeTe and InGeTe monolayers, it does not alone guarantee a non-trivial topological phase.^42^ Because its Z2 invariant is equal to 0, we can conclude that it is not a topological insulator. The conflicting results may be due to the strength of spin–orbit coupling. It is entirely likely despite band inversion, the parities of the occupied electronic states do not lead to a non-trivial topological insulator. The *Z*_2_ calculation for InSnTe seems promising at first, with *Z*_2_ = 1. However, since there are no edge states, we conclude that it is a normal insulator.

## 4. Conclusion

In conclusion, we have predicted a new family of large-gap 2D TIs using first-principles calculations by investigating the geometric, electric and topological properties of each monolayer. In TlP and TlPX_2_ (X = F, Cl, Br, I), TlPF_2_, TlPCl_2_, TlPBr_2_, and TlPI_2_ are promising 2D TIs. In TlAs and TlAsX_2_ (X = F, Cl, Br, I), TlAs, TlAsF_2_, TlAsCl_2_, TlAsBr_2_, and TlAsI_2_ are promising 2D TIs. In GaGeTe, InGeTe, InSnTe, our results show that none of the monolayers were predicted to be promising 2D TIs. Out of these predicted 2D TIs, the band gaps range from 0.03 eV to 0.21 eV. The QSH effect can be detected at room temperature (0.026 eV) for each predicted 2D TI. Our research provides an impressive advance in promising 2D TIs. We firmly believe that these 2D TIs are promising platforms for device application in quantum computers, nanoelectronics, and spintronics.

## Conflicts of interest

There are no conflicts to declare.

## Supplementary Material
